# Phospho-S129 Alpha-Synuclein Is Present in Human Plasma but Not in Cerebrospinal Fluid as Determined by an Ultrasensitive Immunoassay

**DOI:** 10.3389/fnins.2019.00889

**Published:** 2019-08-22

**Authors:** Cristina Cariulo, Paola Martufi, Margherita Verani, Lucia Azzollini, Giordana Bruni, Andreas Weiss, Sean M. Deguire, Hilal A. Lashuel, Eugenia Scaricamazza, Giulia Maria Sancesario, Tommaso Schirinzi, Nicola Biagio Mercuri, Giuseppe Sancesario, Andrea Caricasole, Lara Petricca

**Affiliations:** ^1^Department of Neuroscience, IRBM S.p.A., Rome, Italy; ^2^IRBM Promidis, Rome, Italy; ^3^Laboratory of Molecular and Chemical Biology of Neurodegeneration, School of Life Sciences, Brain Mind Institute, Ecole Polytechnique Fédérale de Lausanne, Lausanne, Switzerland; ^4^Neurology Unit, Department of Systems Medicine, University of Rome “Tor Vergata”, Rome, Italy; ^5^IRCCS Fondazione Santa Lucia, Rome, Italy

**Keywords:** Parkinson’s disease, neurodegeneration, alpha-synuclein, phosphorylation, immunoassay, human, CSF, plasma

## Abstract

Accumulation and aggregation of misfolded alpha-synuclein is believed to be a cause of Parkinson’s disease (PD). Phosphorylation of alpha-synuclein at S129 is known to be associated with the pathological misfolding process, but efforts to investigate the relevance of this post-translational modification for pathology have been frustrated by difficulties in detecting and quantifying it in relevant samples. We report novel, ultrasensitive immunoassays based on single-molecule counting technology, useful for detecting alpha-synuclein and its S129 phosphorylated form in clinical samples in the low pg/ml range. Using human CSF and plasma samples, we find levels of alpha-synuclein comparable to those previously reported. However, while alpha-synuclein phosphorylated on S129 could easily be detected in human plasma, where its detection is extremely sensitive to protein phosphatases, its levels in CSF were undetectable, with a possible influence of a matrix effect. In plasma samples from a small test cohort comprising 5 PD individuals and five age-matched control individuals we find that pS129 alpha-synuclein levels are increased in PD plasma samples, in line with previous reports. We conclude that pS129 alpha-synuclein is not detectable in CSF and recommend the addition of phosphatase inhibitors to plasma samples at the time of collection. Moreover, the findings obtained on the small cohort of clinical plasma samples point to plasma pS129 alpha-synuclein levels as a candidate diagnostic biomarker in PD.

## Introduction

Alpha-synuclein (SNCA) is a protein implicated in the pathogenesis of synucleopathies, of which Parkinson’s disease (PD) is the most prominent example. SNCA is a principal constituent of Lewy bodies, the characteristic histopathological hallmark of PD (Spillantini et al., 1998; Athauda and Foltynie, 2015). In PD, misfolded and aggregated SNCA from post-mortem brain samples is heavily decorated with post-translational modifications [PTMs; (Schmid et al., 2013)], of which phosphorylation at S129 (pS129) is a major component [(Fujiwara et al., 2002); reviewed in Oueslati (2016)]. Given the pathological relevance of the misfolding and aggregation propensity of SNCA in synucleopathies as well as the proposed role of PTMs in SNCA’s misfolding, clearance and aggregation processes, the development of assays capable of detecting and quantifying monomeric and oligomeric SNCA, as well as its post-translationally modified forms (Vicente Miranda et al., 2017), is a key diagnostic and therapeutic focus of translational PD research (reviewed in Anderson et al., 2006; Kasuga et al., 2012; Oueslati, 2016; Parnetti et al., 2019). Based on quantitative mass spectrometry and immunodetection techniques, a variety of assays have been developed to measure SNCA and its species, with several of these having been employed to measure SNCA levels in clinical samples, and in human cerebrospinal fluid (CSF) in particular (reviewed in Atik et al., 2016; Simonsen et al., 2016; Mollenhauer et al., 2019; Parnetti et al., 2019). In general, measured SNCA levels in human CSF vary from hundreds to thousands of pg/ml, with variation owing likely to the nature of the assay and protein standards, intrinsic variability of analyte levels in this matrix as well as to variability in sample collection, handling and storage. In spite of the capacity of CSF (total) SNCA levels to differentiate PD patients from healthy controls, they do not generally correlate with disease progression nor can they differentiate between PD and other synucleopathies such as multiple system atrophy (MSA) or progressive supranuclear palsy (PSP) (Mollenhauer et al., 2011, 2019; Shi et al., 2014). Based on its association with misfolded and aggregated SNCA in PD (Oueslati, 2016), pS129 SNCA represents a potentially attractive candidate stratification and disease progression biomarker (Foulds et al., 2011, 2012, 2013). To date, only two immunoassays detecting the native pS129 SNCA protein, based on the Luminex technology (Wang et al., 2012) and on ELISA (Majbour et al., 2016) have been developed and employed to detect and quantify pS129 SNCA in human CSF. These assays have successfully detected pS129 SNCA in human CSF and established its levels from ca. 60–70 to ca. 220 pg/ml (approx. 12–15% of the total SNCA detected in CSF in the same studies). These assays are being employed to examine correlations between pS129 SNCA levels and pathology, and in a recent study examining pS129 SNCA levels in PD longitudinally using the Luminex pS129 SNCA assay, a non-linear correlation with disease state was observed (Stewart et al., 2015). However, validation of pS129 SNCA detection in CSF in independent laboratories is still required (Parnetti et al., 2019).

Single-molecule counting technology (Singulex Erenna Immunoassay, based on single molecule counting; Singulex assay) has recently been applied to develop ultrasensitive, quantitative immunoassays for the detection of different proteins associated with neurodegenerative pathologies in clinical samples, and in CSF in particular (Savage et al., 2014; Wild et al., 2015). With the objective of further increasing the sensitivity of available SNCA and pS129 SNCA measurements in order to detect and measure pS129 SNCA in CSF, we developed and validated novel assays based on Singulex technology, demonstrating their extreme sensitivity and their specificity using semisynthetic SNCA proteins with or without pS129, cell lysates in combination with expression of SNCA or SNCA bearing an S129A mutation, specific immunodepletion for SNCA, and transfection with known SNCA kinases [GRK1 and PLK2 (Sakamoto et al., 2009; Oueslati et al., 2013; Buck et al., 2015)]. Using these Singulex assays, we then analyzed SNCA and pS129 SNCA levels in human CSF as well as peripheral matrices (plasma) obtained from different sources and investigated the potential influence of matrix-dependent as well as matrix-independent factors on pS129 SNCA and SNCA detection in these clinical matrices. Although we successfully confirmed the detection and quantification of pS129 SNCA and SNCA in plasma and of SNCA in CSF to levels compatible with those previously published, we did not detect pS129 SNCA in CSF despite the extreme sensitivity of our pS129 SNCA immunoassay. Interestingly, we observed that pS129 SNCA levels in plasma are extremely sensitive to endogenous phosphatase inhibition, while SNCA levels are unaffected by addition of phosphatase inhibitors at the time of sample collection, potentially pointing to sample collection/handling as a factor contributing to the variability in reported pS129 SNCA plasma levels. Spike recovery experiments indicated that pS129 SNCA was only partially recoverable in CSF, while no differences in the recovery of SNCA and pS129 SNCA were observed in plasma. This partial loss of pS129 SNCA recovery in CSF was insensitive to pre-treatment of CSF with different denaturing agents or to the addition of phosphatase inhibitors at the time of sample collection. This limits the possibility that pS129 SNCA dephosphorylation, pS129 SNCA degradation or epitope masking due to inter- or intra-molecular interactions contribute to loss of analyte detection/recovery. Instead, SNCA was very efficiently recovered in CSF, suggesting that this matrix specifically interferes with recovery/detection of the S129 phosphorylated SNCA species.

## Materials and Methods

### Antibodies

SNCA and pS129 SNCA Singulex immunoassays were developed using the following antibodies: Covance 4B12 (catalog number #SIG-39730) recognizing the amino acids 103–108 of α-synuclein, Abcam MJFR1 (catalog number #138501) recognizing the amino acids 118–123 of α-synuclein, and Abcam MJF-R13 (8-8) (catalog number #168381) recognizing the phosphorylated Ser129 of α-synuclein. Antibodies against GAPDH and FLAG were distributed by Sigma-Aldrich (catalog #G9545; catalog #F1804). Secondary antibodies used for Western Blotting were Goat-Anti-mouse IgG HRP conjugated (catalog #12–349; Merck) and Goat-Anti-rabbit IgG HRP conjugated (catalog #12–348; Merck). The Alexa-647 labeling on Abcam MJF-R13 (8-8) and Abcam MJFR1 was performed using the Alexa Fluor-647 Monoclonal Antibody Labeling Kit from Thermo Fisher Scientific (catalog #A20186), following the manufacturer’s instructions. Covance 4B12 antibody was conjugated to magnetic particles for Singulex assays, using SMC^TM^ Capture Antibody Labeling Kit (catalog #03-0077-02; Merck) following the manufacturer’s recommendations.

### Semisynthetic Proteins

The semisynthetic SNCA and pS129 SNCA proteins were obtained from Prof. Lashuel (EPFL) and were described before (Paleologou et al., 2010; Fauvet and Lashuel, 2016). Pure Trifluoroacetic acid was added to the lyophilized protein powder for disaggregation and removed by evaporation under fume hood. Proteins were then dissolved in TBS buffer (50 mM Tris 150 mM NaCl) to obtain a final concentration of 20 μM (pH adjusted to 7.2–7.4 using 1M NaOH). Protein solutions were filtered through a 100 kDa membrane (Nanosep Centrifugal Devices 100K Omega-catalog #OD100C34; Pall). Each sample was supplied with 1% Tween-20.

### Plasmids and Constructs

cDNAs encoding human SNCA and human SNCA bearing the phospho-abrogative mutation S to A on residue S129 and cDNAs encoding human PLK2 and human GRK1 (C-terminally FLAG-tagged) were synthesized by Genscript (Piscataway, NJ, United States), quality controlled by DNA sequencing and subcloned into pCDNA3.1. Their expression in mammalian cells was validated by Western Blotting using an anti-FLAG antibody.

### HEK293T Cell Culture and Manipulation

HEK293T cells were cultured and manipulated as described previously (Fodale et al., 2014), harvested 24 h post-transfection and lysed in lysis buffer (TBS, 0.4% Triton X100) supplemented with 1X protease inhibitor cocktail (cOmplete Protease Inhibitor Tablets catalog #11697498001, Roche: inhibit a broad spectrum of serine, cysteine, and metalloproteases, as well as calpains, contain EDTA and both irreversible and reversible protease inhibitors that do not form irreversible complexes with the SH groups of proteins) and 1X phosphatase inhibitors (PhosSTOP catalog #04986837001, Roche: inhibits phosphatase classes such as acid and alkaline phosphatases, as well as serine/threonine (PP1, PP2A, and PP2B) and tyrosine protein phosphatases (PTP) in bacterial, mammalian, yeast, and plant extracts).

### Western Blotting

For Western Immunoblotting, samples were denatured at 95°C in 4X Loading Buffer (125 mM Tris-HCl pH 6.8, 6% SDS, 4 M urea, 4 mM EDTA, 30% Glycerol, 4% 2-Mercaptoethanol and Bromophenol Blue) and loaded on NuPAGE 4–12% Bis-Tris Gel (catalog #WG1402BOX; Thermo Fisher Scientific). Proteins were transferred on PVDF membrane (catalog #162–0177; Bio-Rad Laboratories) using wet blotting. After fixing in 0.4% paraformaldehyde/0.4% sucrose solution and blocking with 5% non-fat milk in TBS/0.1% Tween-20, primary antibody incubation was carried out overnight at 4°C and secondary antibody incubations for 1 h at room temperature. Protein bands were detected using chemiluminescence substrate (Supersignal West Femto Maximum catalog #3406; Supersignal West Pico Maximum catalog #34087; Thermo Fisher Scientific) on Chemidoc XRS + (Bio-Rad Laboratories).

### Human Samples

Commercial human CSF and commercial human plasma were acquired at BioIVT. Informed consent was obtained from individuals admitted to the Neurological Clinic of University of Rome Tor Vergata between May 2015 and November 2016 (age 70.09 + 6.49, male/female ratio 1.4). CSF was obtained as described in Sancesario et al. (2010). All subjects underwent a complete diagnostic work-up with medical record, neurologic examination, blood sample, electroencephalogram (EEG), brain magnetic resonance imaging (MRI), and lumbar puncture (LP). Samples of 1 ml of CSF were collected in sterile polypropylene tubes containing 100 μl of protease and phosphatase inhibitors cocktail (catalog #11697498001 and catalog #04986837001; Roche). CSF was then centrifuged for 10 min at 2.000 rpm at 4°C and stored in aliquots of 500 μl at −80°C within 60 min of completion of the lumbar puncture. Plasma was obtained by routine phlebotomy using 5 ml plastic serum and K2EDTA collection tubes. Samples were immediately mixed with 500 μl of protease and a phosphatase inhibitor cocktail. After being centrifuged at 3,500 rpm for 10 min at 4°C, aliquots were stored at −80°C until analysis. The Local Ethics Committee approved the study procedures.

### Immunoprecipitation

Immunoprecipitation was performed using 50 μl of Dynabeads^®^ Protein G (catalog #10004D; Thermo Fisher Scientific) following the manufacturer’s instructions and using 10 μg of an a-synuclein-specific antibody (catalog #S5566; Sigma), different from the ones employed in the Singulex assays or 10 μg of an unrelated antibody (Anti-Glial Fibrillary Acidic Protein GFAP; catalog #G3893; Sigma). The supernatant (immunodepleted sample) was analyzed by Singulex assays.

### Singulex Assays

A total of 50 μL/well of dilution buffer (6% BSA, 0.8% Triton X-100, 750 mM NaCl, and complete protease inhibitor) was added to a 96-well plate (catalog #P-96-450V-C; Axygen), and 15 μl of artificial cerebral spinal fluid (0.3 M NaCl; 6 mM KCl; 2.8 mM CaCl2-2H2O; 1.6 mM MgCl2-6H20; 1.6 mM Na2HPO4-7H2O; 0.4 mM NaH2PO4-H2O) supplemented with complete protease inhibitors and 10% Tween-20 was added to 135 μl of the sample to be tested in order to obtain a final volume of 150 μl at 1% Tween-20 concentration. Then, 25 μg of 4B12 #SIG-39730 antibody per mg of magnetic particles were coupled according to Erenna Capture Kit manufacturer’s instructions (catalog #03-0077-02). The labeled magnetic particles were resuspended in Coated Bead Buffer (provided with the labeling kit catalog #03-0077-02) at 10 mg/mL. Finally, 100 μL/well of the Covance 4B12 antibody coupled with magnetic particles (diluted in Erenna Assay buffer catalog #02-0474-00 (Merck) to a final concentration of 5 μg/well) was added to the assay plate, and incubated for 2 h at room temperature under orbital shaking. The beads were then washed with Erenna System buffer (catalog #02-0111-00; Merck) and resuspended with 20 μL/well of labeled detection antibodies (Abcam MJF-R13 (8-8) for pS129 and Abcam MJFR1 for total SNCA) in Erenna Assay buffer. The detection antibodies were custom-labeled with Alexa-647 fluorophore at cisbio, and stored as 0.5 mg/mL. The final working concentration was 10 ng/well. The plate was incubated for 1 h at room temperature under shaking. After washing, the beads were resuspended and transferred in a new 96-well plate. A total of 10 μL/well of Erenna buffer B (catalog #02-0297-00; Merck) was added to the beads for elution and incubated for 5 min at room temperature under orbital shaking. The eluted complex was magnetically separated from the beads and transferred in a 384-well plate (Nunc catalog #264573; Sigma) where it was neutralized with 10 μL/well of Erenna buffer D (catalog #02-0368-00; Merck). Finally, the 384-well plate was heat-sealed and analyzed with the Erenna Immunoassay System.

### Data Analysis

Semisynthetic SNCA and pS129 SNCA proteins were used as reference standards for SNCA and pS129 SNCA Singulex assays, respectively. The standard curves were generated by serial dilution at 1:2.5 of the first concentration point (16000 pg/ml for SNCA Singulex assay and 2560 pg/ml for pS129 SNCA Singulex assay). The unknown SNCA and pS129 SNCA sample concentrations (pg/ml) were obtained through a back-calculation on the reference standards by interpolation of the signals for each readout. Graphs were generated and statistical analysis was performed using the software GraphPad Prism 6. In all statistical analyses, degrees of significance are as follows: ^∗^*p* < 0.05, ^∗∗^*p* < 0.01, ^∗∗∗^*p* < 0.005, and ^****^p < 0.001.

## Results

### Development of Singulex Erenna Immunoassays (Singulex Assays) for SNCA and pS129 SNCA

**FIGURE 1:**
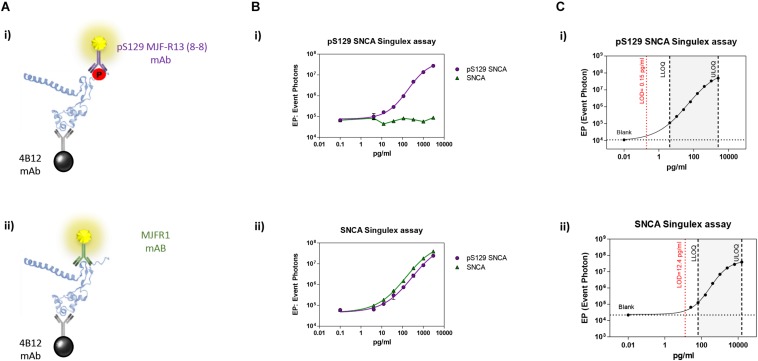
Validation and characterization of Singulex assays for the detection of pS129 SNCA and SNCA using purified, semisynthetic full length SNCA proteins. **(A)** Antibodies selected for the pS129 SNCA Singulex assay **(i)** and SNCA Singulex assay **(ii)**. **(B)** Specificity of the pS129 SNCA and SNCA Singulex assays. **(i)** Serial dilutions of semisynthetic pS129 SNCA and SNCA analyzed with the pS129 SNCA Singulex assay and **(ii)** with the SNCA Singulex assay. **(C)** Characterization of the pS129 SNCA **(i)** and SNCA **(ii)** and assay performance evaluation of Singulex assays using semisynthetic pS129 or SNCA proteins.

Immunoassay development relies on the availability of antibodies capable of specifically recognizing the epitopes of interest, and of the purified antigen protein in order to assess assay specificity and sensitivity in a controlled context. As only a fraction of the total steady-state pool of SNCA may be phosphorylated in a biological sample at any one time, we turned to one of the most sensitive immunoassay platforms available (Todd et al., 2007), essentially a quantitative fluorescent sandwich immunoassay coupled to single-molecule counting technology (Singulex Erenna immunoassay). This technology was recently employed to develop ultrasensitive immunoassays for the detection of oligomeric amyloid beta (Savage et al., 2014), mutant Huntingtin (Wild et al., 2015) as well as Huntingtin phosphorylated at residue T3 (Cariulo et al., 2017). Two antibodies are required (one for capture and one for detection; Supplementary Figure S1A). Importantly, an immunoassay for the measurement of pS129 SNCA levels needs to be coupled to a companion immunoassay for the measurement of overall SNCA levels to allow for normalization of phosphorylated analyte levels. The two immunoassays (pS129 SNCA-specific and total SNCA-specific) need to be ideally based on the same capture antibody in order to enable meaningful associations between pS129 SNCA and total SNCA levels (Supplementary Figure S1A). Full length SNCA and pS129 SNCA proteins of well characterized purity and quality (Lu et al., 2011; Fauvet and Lashuel, 2016) were employed as a source of purified antigen for assay development efforts. Initially, multiple monoclonal antibodies (mAbs) with reported specificity for SNCA proteins were commercially sourced and profiled by Singulex assay through a combinatorial, comparative testing approach (Supplementary Figure S1B). In this analysis, the behavior of each mAb as a capture IgG in combination with every other mAb as a detection IgG was examined under identical conditions on a serial dilution curve of SNCA proteins. For this comparison, the ratio of the signal obtained in the presence of the analyte to that obtained in the absence of the analyte (signal/background) was used as a measure of the relative sensitivity and specificity of the different mAb combinations. This combinatorial analysis (Supplementary Figure S2) allowed the identification of a mAb (4B12) which could be used as a capture IgG to for sandwich detection of both SNCA and pS129 SNCA proteins when coupled to relevant detection mAbs (mAb MJFR1 and mAb MJF-R13 8-8, respectively), therefore satisfying the design envisaged for the assay (a single mAb for capturing SNCA protein and two separate mAbs for detecting total SNCA and pS129 SNCA; Figure 1A). Following optimization of capture and detection mAb concentrations, a full concentration response analysis was performed on purified SNCA and pS129 SNCA proteins to determine the specificity of the immunoassay (pS129 Singulex assay: 4B12/MJF-R13 8-8 and SNCA Singulex assay: 4B12/MJFR1; Figure 1B). A detailed technical validation of the 4B12/MJF-R13 (8-8) and 4B12/MJFR1 Singulex assays was performed to establish sensitivity, performance, precision, stability and accuracy [according to Armbruster and Pry (2008); Supplementary Figures S3, S4 and Figure 1C] using purified, semisynthetic SNCA and pS129 SNCA proteins. Assay performance was assessed resulting in a limit of detection (LOD), lower limit of quantification (LLOQ) and upper limit of quantification (ULOQ) of 0.15, 4.19 and 2560 pg/ml, respectively, for the pS129 SNCA Singulex assay (Figure 1C-i) and 12.4, 65.5 and 16000 pg/ml, respectively, for the SNCA Singulex assay (Figure 1C-ii). The limits of detection of the SNCA and pS129 SNCA Singulex assays are comparable to the most sensitive amongst published SNCA and pS129 SNCA immunoassays, such as those based on Luminex technology [(Wang et al., 2012); 9 pg/ml for SNCA and pS129 SNCA] and on AlphaLISA [(Landeck et al., 2016); 3.7 pg/ml for SNCA and 1.1 pg/ml for pS129 SNCA], and were significantly more sensitive than assays based on ELISA [(Majbour et al., 2016); 50 pg/ml for SNCA and 20 pg/ml for pS129 SNCA].

**FIGURE 2:**
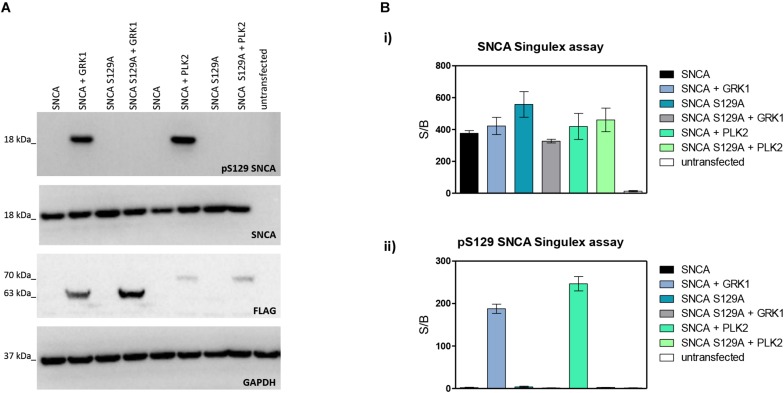
Validation and characterization of Singulex assays for the detection of pS129 SNCA and SNCA using HEK293T cells expressing SNCA in the presence or absence of the S129A mutation or of an SNCA kinase. **(A)** Western blotting of HEK293T cells transfected with expression plasmids encoding human SNCA or its S129A mutant, in the presence or absence of expression plasmids encoding FLAG-tagged kinases GRK1 and PLK2. SNCA expression, phosphorylation of SNCA on S129 and kinase expression were properly detected using specific antibodies. **(B)** **(i)** SNCA levels detected in HEK293T demonstrates correct overexpression of SNCA proteins irrespective of S129 status (S129A or SNCA kinase co-expression). Co-expression of GRK1 and PLK2 with SNCA does not result in a significant increase in SNCA levels. **(ii)** pS129 SNCA levels detected in the same samples demonstrates the signal is sensitive to S129 status, as the presence of the S129A mutation abolishes the effect of SNCA kinases on pS129 SNCA signal. Co-expression of GRK1 and PLK2 with SNCA results in a strong increase in pS129 SNCA levels.

Next, the SNCA and pS129 SNCA Singulex assays were interrogated for their capacity to specifically detect their respective analytes when these are expressed in a complex biological matrix. HEK293T cells were chosen as an experimental cell line as different groups previously reported successful overexpression of SNCA and modulation of its S129 phosphorylation status in these cells (Sakamoto et al., 2009; Landeck et al., 2016). Expression constructs for human SNCA or its S129A mutant (impaired for S129 phosphorylation) were transfected into HEK293T cells in the presence or absence of selected kinases known to affect SNCA phosphorylation at residue S129 (reviewed in Tenreiro et al., 2014). As shown in Figure 2A, HEK293T cells transfected with plasmids encoding human cDNAs for SNCA or S129A SNCA produced significant amounts of SNCA protein, which could be effectively phosphorylated on S129 by SNCA kinases GRK1 and PLK2 as detected by specific antibodies in Western blotting. In the presence of the S129A mutation, GRK1 and PLK2 failed to induce SNCA phosphorylation on S129, as expected. The same cell lysates were then subjected to analysis using the pS129 SNCA and SNCA Singulex assays (Figure 2B). The SNCA Singulex assay detects comparable levels of SNCA in all samples overexpressing the SNCA construct, with little variations associated with kinase co-expression or presence of the S129A mutation (Figure 2Bi). Coherent with specific pS129 SNCA detection, the pS129 SNCA Singulex assay detects high levels of pS129 SNCA in lysates expressing SNCA in the presence of GRK1 or PLK2, with very low signal in all samples expressing S129A, probably due to the endogenous SNCA (3Bii). Interestingly, even at relatively lower expression levels, PLK2 produced a robust increase in pS129 SNCA levels, comparable to GRK1 (Figures 2A,B). In these experiments, which were intended to produce positive controls for pS129 SNCA modulation, we did not observe the reported modulation of total SNCA levels by PLK2 (Oueslati et al., 2013; Kofoed et al., 2017), likely because of low PLK2 expression levels in our experiments (Figure 2A). The SNCA and pS129 SNCA signals detected by the Singulex assays under overexpression conditions are sensitive to immunodepletion with a specific anti-SNCA mAb (Supplementary Figures S5A,B, respectively) thus further validating the specificity of the Singulex assays.

### SNCA and pS129 SNCA Detection in Human Cerebrospinal Fluid

**FIGURE 3:**
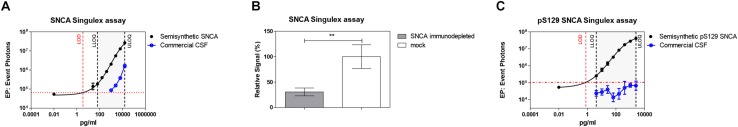
Detection of SNCA and pS129 SNCA in human CSF (commercial source) using the Singulex assays. **(A)** SNCA levels in a dilution series of commercial CSF demonstrating parallelism with a serial dilution of SNCA semisynthetic protein. **(B)** Specificity by immunodepletion of SNCA in the CSF sample using anti-SNCA mAb (S5566-Sigma). Means and SDs of three independent experiments. *T*-test (*p* < 0.001). **(C)** pS129 SNCA signal is undetectable in the same commercial CSF sample using the pS129 SNCA Singulex assay.

**FIGURE 4:**
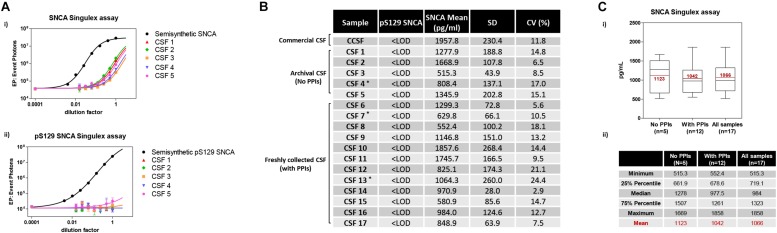
Detection and quantification of SNCA and pS129 SNCA in human CSF (clinical source) using the Singulex assays. **(A)** Analysis of SNCA **(i)** and pS129 SNCA **(ii)** levels in 5 archival CSF samples of clinical origin (CSF 1–5; ^∗^PD individuals). **(B)** Quantification of pS129 SNCA (undetectable) and SNCA levels in the 5 archival clinical CSF samples (CSF 1–5) and in an additional 12 freshly collected clinical CSF samples treated with phosphatase inhibitors (PPIs) at the time of collection (CSF 6–17; ^∗^PD individuals). **(C)** (i) Box plot illustrating SNCA levels obtained using the SNCA Singulex assay in the clinical CSF samples, subdivided per group (left: archival CSF samples not treated with PPIs; center: freshly collected, PPIs treated clinical CSF samples; right: all clinical CSF samples). Mean values of SNCA (pg/ml) are in line with previous findings. **(ii)** Table illustrating statistical distribution of measured values (minimum and maximum, quartiles and mean).

**FIGURE 5:**
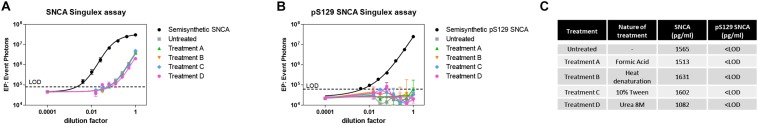
Effects of different denaturing/solubilizing treatments on pS129 SNCA and SNCA detection in CSF. **(A)** Effects of denaturating conditions employing formic acid (treatment A), heat denaturation at 95°C for 5 min (treatment B), detergent 10% Tween (treatment C) and Urea 8M (treatment D) on SNCA detection in commercial CSF. Treatments do not affect SNCA detection. Curves obtained on serial dilutions of CSF. **(B)** Effects of same treatments described in A. on pS129 SNCA detection in commercial CSF. pS129 SNCA levels remain undetectable in all tested conditions. **(C)** Summary and quantification of **(A,B)**.

**FIGURE 6:**
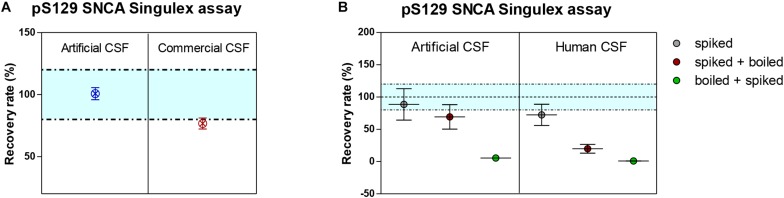
Recovery of spiked semisynthetic pS129 SNCA protein in artificial, commercial and human clinical CSF. **(A)** Recovery of pS129 SNCA in artificial CSF is complete, while it is only partial in commercial CSF, indicating a matrix effect. **(B)** Recovery of pS129 SNCA in human clinical CSF is only partial and as demonstrated for endogenous detection of pS129 SNCA, denaturation (by heat) does not allow for complete recovery of spiked pS129 SNCA signal.

Given the interest in quantifying pS129 SNCA and SNCA in clinical samples, and in CSF in particular (Foulds et al., 2012, 2013; Wang et al., 2012; Stewart et al., 2015; Majbour et al., 2016, 2017), we applied the new Singulex assays to measure SNCA and pS129 SNCA levels in CSF. Previous studies employing an ELISA detection platform detected ca. 1600 pg/ml of SNCA and ca. 220 pg/ml pS129 SNCA in CSF (Majbour et al., 2016), while an independent study employing a Luminex detection platform reported ca. 500 pg/ml SNCA and ca. 70 pg/ml pS129 SNCA (Wang et al., 2012) in the same matrix. Another ELISA-based study reported CSF SNCA and pS129 SNCA levels in the range of 1800–3800 pg/ml and 3600–7140 pg/ml, respectively (Foulds et al., 2012). In commercially sourced CSF we successfully detected SNCA (Figure 3A) in a specific fashion (Figure 3B) but were unable to successfully detect pS129 SNCA (Figure 3C), where the signal was under the limit of detection of the assay (0.15 pg/ml). Having observed no pS129 SNCA signal in commercially sourced CSF, we reasoned that sample collection and/or handling may be a potential factor impacting the ability to detect the analyte in this sample. We therefore decided to analyze pS129 SNCA levels in archival (*N* = 5) CSF samples collected as described (Liguori et al., 2017) and then in freshly collected CSF samples with (*N* = 12) from individuals that were supplemented with phosphatase inhibitors (PPIs). As shown in Figure 4A, pS129 SNCA and SNCA levels measured using the two Singulex assays in five archival CSF samples produced robust detection of SNCA (Figure 4Ai), while pS129 SNCA levels were undetectable (Figure 4Aii). Analysis in additional, freshly collected CSF samples (*N* = 12; Figure 4B) produced comparable results (Figure 4C). SNCA detection in these CSF samples was essentially uninfluenced by the addition of a PPIs cocktail at the time of collection (Figure 4C) and was in the range of 500–2000 pg/ml (Figure 4Cii), which is consistent with published measurements (Wang et al., 2012; Majbour et al., 2017). Therefore, pS129 SNCA levels remained undetectable in freshly collected, PPIs-treated CSF samples as observed for commercially sourced CSF (Figure 4B). We conclude that the lack of pS129 SNCA detection by our pS129 SNCA Singulex assay is independent of factors associated with standard collection and/or handling of CSF samples. As detection of pS129 SNCA in CSF samples was previously reported using other immunoassay platforms (Foulds et al., 2012; Wang et al., 2012; Majbour et al., 2016) we hypothesized that detection by our pS129 SNCA Singulex assay in CSF may be influenced by epitope masking events, such as those associated with analyte conformation or intra- and inter-molecular protein-protein interactions, which may impede epitope detection by the mAbs employed in our assay. To address this issue we subjected CSF samples to a variety of physico-chemical denaturing treatments prior to analysis with our pS129 SNCA Singulex assay. As shown in Figures 5A,C, levels of SNCA in CSF were unaffected by treatment with different denaturing conditions, indicating that sample integrity was maintained. In spite of treatment with different denaturing conditions, pS129 SNCA levels in CSF remained at or below the limit of detection (Figures 5B,C). These experiments suggested that either levels of pS129 SNCA in CSF are below the limit of detection of our assay (0.15 pg/ml), or that this matrix is unpermissive for pS129 SNCA detection using our assay. To address this latter hypothesis, we performed spike recovery experiments using purified semisynthetic pS129 SNCA in artificial CSF (ACSF) or in commercially sourced CSF (Figure 6). As shown in Figure 6A, spiked pS129 SNCA protein was fully recovered from ACSF, indicating that no matrix effects are present to influence pS129 SNCA measurement. On the other hand, spiked pS129 SNCA was inefficiently recovered from CSF (ca. 70% recovery), suggesting that one or more factors in this matrix interfere with adequate detection of pS129 SNCA. Note that we had already excluded the influence of phosphatase activity (at least the activity of phosphatases which can be inhibited by standard phosphatase inhibitors cocktails) in CSF as a potential interfering factor (Figure 4). We also excluded that a temperature/denaturation-sensitive factor (such as a non-covalent intra- or inter-molecular interaction) may interfere with detection of spiked pS129 SNCA in CSF (Figure 6B) because of the inability of various denaturing treatments to recover endogenous pS129 SNCA signal in CSF (Figures 5B,C).

### SNCA and pS129 SNCA Detection in Human Plasma

**FIGURE 7:**
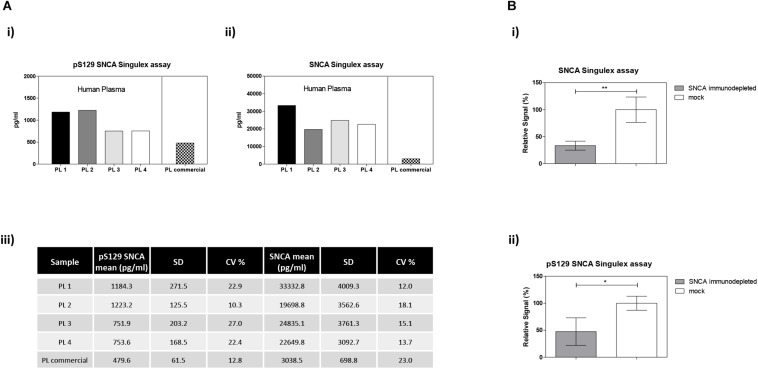
Detection and quantification of pS129 SNCA and SNCA in human plasma using the Singulex assays. **(A)** Quantification of pS129 SNCA (i) and SNCA (ii) in 4 clinically obtained and 1 commercial sourced plasma samples. **(iii)** Table indicating measured values (pg/ml) of pS129 SNCA and SNCA in human plasma. **(B)** Specificity of SNCA and pS129 SNCA signals in plasma samples by immunodepletion with anti-SNCA mAb (S5566-Sigma) or with the unrelated GFAP mAb (G3893-Sigma). Means and SDs of 3 independent experiments. *T*-test (*p* < 0.05).

**FIGURE 8:**
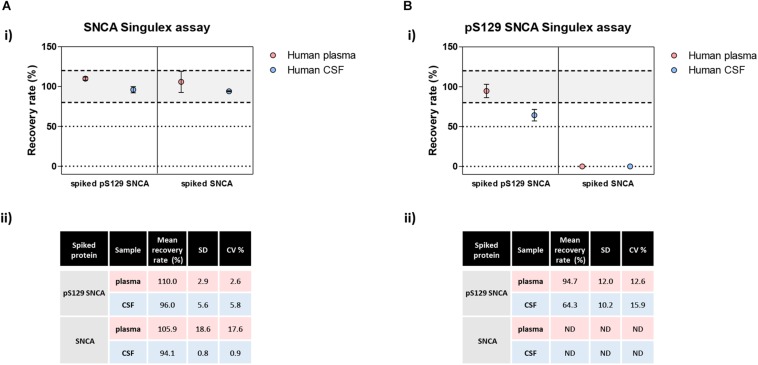
Analysis of matrix effects in pS129 SNCA and SNCA detection in human plasma and CSF. **(A)** Detection of spiked semisynthetic pS129 SNCA and SNCA proteins in two human clinical plasma and two CSF samples using SNCA Singulex assay. (i) Recovery of pS129 SNCA and SNCA proteins in human plasma and CSF samples is complete, as measured by SNCA Singulex assay. (ii) Table indicating means of recovery rates, SD and CV% of human plasma and CSF samples from two individuals. **(B)** Detection of spiked semisynthetic pS129 SNCA and SNCA proteins in two human clinical plasma and two CSF samples using pS129 SNCA Singulex assay. (i) Recovery of pS129 SNCA in human plasma is complete, while it is only partial in human CSF as measured by pS129 SNCA Singulex assay, indicating a matrix effect. Semisynthetic SNCA protein is not detected by pS129 SNCA Singulex assay as expected. (ii) Table indicating mean of recovery rates, SD and CV% of human plasma and CSF samples from two individuals.

**FIGURE 9:**
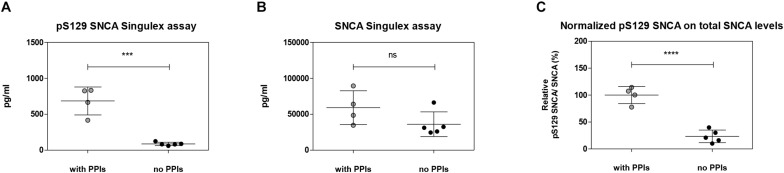
Effects of the inhibition of endogenous phosphatase activity in plasma on pS129 SNCA. **(A)** Quantification of pS129 SNCA in human plasma samples in the presence or absence of phosphatase inhibitors cocktail (PPIs) at the time of sample collection using pS129 SNCA Singulex assay. **(B)** Quantification of SNCA in human plasma samples in the presence or absence of PPIs at the time of sample collection using SNCA Singulex assay. **(C)** Estimation of relative pS129 SNCA in human plasma samples. pS129 SNCA levels are normalized for SNCA levels. Mean and SD of 4 (with PPIs) and 5 (no PPIs) human plasma samples. *T*-test (*p* < 0.001).

**FIGURE 10:**
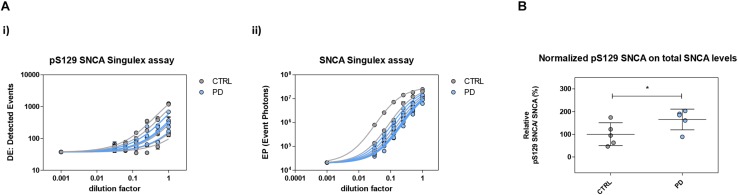
Plasma pS129 SNCA levels in control and PD individuals. **(A)** Assessment of pS129 SNCA **(i)** and total SNCA **(ii)** levels in a test cohort comprising 5 PD individuals and 5 age-matched control individuals. The signals obtained with each immunoassay from serial dilutions of plasma samples from each individual are shown. **(B)** pS129 SNCA levels (normalized for total SNCA levels) in each of the plasma samples from the 5 control and PD individuals. Mean and SD are shown. *T*-test (*p* < 0.05).

The possibility of further CSF-matrix specific effects or of extremely low pS129 SNCA levels in CSF was explored by applying our pS129 SNCA Singulex assay to another commonly sampled clinical matrix, namely human plasma, where datasets are available for both SNCA and pS129 SNCA levels (El-Agnaf et al., 2003; Foulds et al., 2011; Gorostidi et al., 2012; Foulds et al., 2013; Koehler et al., 2015). Plasma pS129 SNCA levels were found to be higher in PD samples than in samples from control individuals (Foulds et al., 2011, 2013; Parnetti et al., 2019), while total SNCA plasma levels appear to be a less reliable diagnostic biomarker for PD, likely because of the contribution of red blood cell-derived SNCA in this matrix (Parnetti et al., 2019). We therefore measured pS129 SNCA and SNCA levels in commercially sourced and freshly collected plasma samples, demonstrating successful detection of both analytes in this matrix (Figure 7A). The detected SNCA and pS129 SNCA signals detected in plasma were specific as demonstrated by immunodepletion experiments (Figure 7B). In human plasma, we measured SNCA levels in the range of thousands of pg/ml (Figure 7Aiii), which is in line with what has been previously reported by others (Foulds et al., 2011, 2013; Shi et al., 2014; Ishii et al., 2015; Koehler et al., 2015). pS129 SNCA levels in the plasma samples we analyzed were in the range of 1000 pg/ml. We investigated the recovery of spiked pS129 SNCA and SNCA proteins in human plasma and, for comparison, in CSF samples from two individuals in parallel. The recovery of spiked pS129 SNCA and SNCA proteins measured using the SNCA Singulex assay was complete in both matrices, indicating that SNCA signal is present and free from matrix-effects (Figure 8A). Conversely, efficient recovery of pS129 SNCA protein measured using pS129 SNCA Singulex assay was possible only in plasma (Figure 8B), confirming a CSF matrix effect on SNCA S129 phosphorylation detection. Interestingly, a robust increase in pS129 SNCA levels in plasma was detected when samples were supplemented with a cocktail of PPIs at the time of collection (Figure 9A). Significantly, SNCA levels in plasma were not affected by phosphatase inhibition (Figure 9B), suggesting that accurate pS129 SNCA measurement in plasma requires careful sample processing to inhibit endogenous phosphatase activity in this matrix. This is particularly relevant when measures of relative plasma SNCA S129 phosphorylation are made, where pS129 SNCA values are normalized for SNCA values, as inappropriate sample handling may lead to underestimation of pS129 SNCA phosphorylation (Figure 9C). Having established that the total SNCA and pS129 SNCA single molecule counting immunoassays can efficiently and specifically detect their respective analytes in human plasma, we analyzed plasma samples from a small test cohort comprising 5 PD individuals and 5 age-matched control individuals. The signals obtained with the pS129 SNCA and total SNCA in plasma samples from control and PD individuals are presented in Figures 10Ai,ii, respectively. As presented in Figure 10B, normalized pS129 SNCA levels were increased in PD plasma samples relatively to samples from control individuals. These results, though obtained on a small cohort of samples, are fully consistent with previously reported findings, pointing to plasma pS129 SNCA levels as a candidate diagnostic biomarker in PD (Foulds et al., 2013; Parnetti et al., 2019).

## Discussion

The development of disease modifying therapeutics for PD and related disorders requires the concurrent development of suitable biomarkers to support primary diagnosis, monitor disease progression, enable patient stratification and interrogate the efficacy of therapeutics in the clinic. SNCA accumulation and misfolding, as a result of genetic mutations or (much more frequently) owing to as yet poorly defined causes is believed to be a primary cause of pathology in synucleopathies (Wong and Krainc, 2017). Clearly, the possibility to accurately detect and quantify levels of SNCA and its pathology-relevant variants (such as pS129 SNCA) and conformers (such as oligomeric SNCA) represents a significant benefit to therapeutics development efforts. Several groups have reported the development of quantitative immunoassays for the detection and quantification of SNCA, pS129 SNCA and oligomeric SNCA in clinical samples, notably CSF and plasma (Bidinosti et al., 2012; Foulds et al., 2012, 2013; Gorostidi et al., 2012; Wang et al., 2012; Koehler et al., 2015; Stewart et al., 2015; Majbour et al., 2016; Parnetti et al., 2019). Specifically, pS129 SNCA represents a particularly interesting SNCA modification as it is enriched in aggregated and misfolded SNCA in PD tissues (reviewed in Oueslati, 2016), it exacerbates pathology and induces neuronal dysfunction in mice (Karampetsou et al., 2017) and its plasma levels are increased in PD (Foulds et al., 2011, 2013). The reported absolute concentration of SNCA analytes is often very variable, which is likely the result of the application of different assays (e.g., different antibodies, different SNCA proteins/peptides for the generation of standard curves, different methodological protocols and equipment), biological variability in analyte concentration in the chosen matrices as well as variation in sample collection, handling and storage protocols. For instance, levels of total SNCA and pS129 SNCA in control human CSF were reported to be 1870 ng/ml and 3580 ng/ml by one group [averages, respectively (Foulds et al., 2012)], while others reported much lower values, and specifically of ca. 0.520 ng/ml and 0.068 ng/ml [averages, respectively (Wang et al., 2012; Hong et al., 2010)] or 1.1 ng/ml and 0.082 ng/ml [averages, respectively (Majbour et al., 2017)]. A capture step-independent, homogeneous immunoassay based on TR-FRET technology measured SNCA levels in human CSF at 0.53 ng/ml [average (Bidinosti et al., 2012)]. Thus, while a majority of studies appear to converge on values of CSF SNCA in the low ng/ml range, only a few and discordant studies provide estimates of CSF pS129 SNCA levels, which require independent validation (Parnetti et al., 2019). With an interest to provide an independent measurement of pS129 SNCA in human CSF, we applied a quantitative fluorescent sandwich immunoassay coupled to single-molecule counting technology to develop and validate ultrasensitive novel immunoassays for the detection of SNCA and its post-translationally modified variant phosphorylated at S129. We previously successfully applied this technology for the detection of another neurodegenerative disease-associated protein, namely mutant huntingtin and a T3 phosphorylated form in preclinical and clinical samples (Wild et al., 2015; Cariulo et al., 2017). Importantly, in order to develop the SNCA and pS129 SNCA Singulex assays, we employed semisynthetic, full length SNCA proteins of known purity and concentration (Fauvet and Lashuel, 2016) to minimize the possibility of experimental artifacts associated with the use of shorter SNCA peptide sequences as protein standards for assay development and characterization. Additionally, we validated the Singulex assays for SNCA and pS129 SNCA detection in biological samples using a human cell line expressing SNCA encoding constructs under conditions known to affect their pS129 SNCA status, such as the introduction of an S129A mutation and/or co-expression of SNCA kinases. Using these Singulex assays, we successfully detected SNCA in human CSF samples of different origin, quantifying its levels at 1066 pg/ml (average; ± 419.5 pg/ml), which is compatible with most studies (Hong et al., 2010; Bidinosti et al., 2012; Wang et al., 2012; Majbour et al., 2017). However, pS129 SNCA was undetectable in CSF using our pS129 SNCA Singulex assays, contrary to previous studies (Wang et al., 2012; Majbour et al., 2017). This discrepancy could not be ascribed to insufficient sensitivity as our assay has a detection limit of 0.15 pg/ml, well above CSF pS129 SNCA levels reported by others (Foulds et al., 2012; Wang et al., 2012; Majbour et al., 2016), or to factors sensitive to denaturants (such as conformational effects or intra-/inter-molecular interactions) as well as sample handling and storage. Interestingly, we observed a modest matrix-dependent interference in pS129 SNCA detection in CSF during spike-recovery experiments (not observed with SNCA), suggesting that CSF contains a denaturation-resistant factor or factors which specifically interfere with the detection of S129 phosphorylated SNCA protein. When we interrogated human plasma [as another clinically relevant matrix which has been previously used to detect and quantify SNCA and pS129 SNCA (El-Agnaf et al., 2003; Foulds et al., 2011, 2013; Gorostidi et al., 2012; Koehler et al., 2015)] with our Singulex assays, both SNCA and pS129 SNCA were successfully and specifically detected and quantified (20711 ± 11107 pg/ml and 878.5 ± 317.4 pg/ml, respectively). Importantly, when a small test cohort of plasma samples from control and PD individuals was analyzed using the novel immunoassays reported here, significantly increased pS129 SNCA levels were observed in PD plasma, in line with previous findings (Foulds et al., 2013). In the course of these experiments, we observed a significant sensitivity of plasma pS129 SNCA levels (but not of SNCA levels) to inhibition of endogenous phosphatase activity, with pS129 SNCA levels which were at least 10-fold higher in plasma samples supplemented with a phosphatase inhibitor cocktail immediately after collection. This is an important observation which needs to be taken into consideration in future experiments aimed at measuring pS129 SNCA in plasma and suggests sample collection/handling as a crucial contributor toward variability in reported pS129 SNCA plasma measurements. Only one study has previously measured plasma SNCA and pS129 SNCA levels in the same sample set (Foulds et al., 2013), reporting values of 1222 ± 2233 ng/ml and 143 ± 531 ng/ml, respectively, and suggesting increased plasma pS129 SNCA levels as a potentially useful diagnostic biomarker in PD. Although the absolute plasma SNCA and pS129 SNCA values reported by the present manuscript and others (Gorostidi et al., 2012; Foulds et al., 2013) are not concordant (with a likely variable represented by endogenous phosphatases effects on plasma sample measurements, see above), SNCA values detected by our Singulex assay in human plasma are compatible with plasma SNCA values reported by several groups (Shi et al., 2014; Ishii et al., 2015; Koehler et al., 2015) where average plasma SNCA levels were estimated in the range of 10–30 ng/ml. We conclude that the SNCA and pS129 SNCA Singulex assays we described in the present work can successfully detect SNCA and pS129 SNCA in a clinically relevant sample, namely human plasma, and can reproduce published data indicating increased pS129 SNCA levels in PD plasma samples (Foulds et al., 2013). Moreover, as pS129 SNCA is reported to be enriched in plasma of PD patients and to associate with aggregated/misfolded SNCA in Lewy bodies (Parnetti et al., 2019), our pS129 SNCA assay can be meaningfully applied to identify and validate pS129 SNCA modifiers employed as potential therapeutics for PD, serving as a non-invasive, potential wet biomarker in preclinical studies and clinical trials aimed at lowering pS129 SNCA levels with the objective of modulating SNCA oligomerization/aggregation. Compared to existing SNCA and pS129 SNCA assay pairs, these novel pS129 SNCA and SNCA assays, with an LOD of 0.15 pg/ml and LOD 12.4 pg/ml, respectively, are more sensitive than formerly published immunoassays [(Majbour et al., 2016), LODs of 50 and 20 pg/ml, respectively (Foulds et al., 2011), LODs of 2 and 30 pg/ml, respectively]. The simultaneous use of several sensitive SNCA and pS129 SNCA immunoassays, based on different mAbs on the same cohorts of phosphatase cocktail inhibitor-treated plasma samples from control and PD individuals, may allow a definitive answer to the utility of blood pS129 SNCA levels as a PD biomarker.

We also successfully detected SNCA in CSF and estimate levels compatible with published work. The fact that our pS129 SNCA Singulex assay does not detect pS129 SNCA in CSF contrasts with previous findings where pS129 SNCA was detected in the range of 70–200 pg/ml (Wang et al., 2012; Majbour et al., 2016), well above the detection limit of our assay. A possible reason for this discrepancy may be a matrix-specific effect of CSF for pS129 SNCA detection, which has not been described in previous reports of pS129 SNCA detection in CSF (Wang et al., 2012; Majbour et al., 2016) and therefore needs to be assay-specific. However, the relatively minor (<50%) influence of this effect on pS129 SNCA signal recovery, the extreme sensitivity of the assay and the levels of pS129 SNCA previously described in CSF make this an unlikely single reason for a lack of pS129 SNCA signal in this clinical matrix. Another possible reason may be the activity of proteases or phosphatases specifically influencing pS129 SNCA in CSF. Although we have supplemented CSF samples with protease inhibitors (a standard procedure) or with phosphatase inhibitors (this work), we cannot exclude that a protease and/or phosphatase which is resistant to such inhibitors is present in CSF and contributes to the lack of pS129 SNCA signal and to loss of recovery in spiked pS129 SNCA in this matrix. Likewise, we cannot exclude that pS129 SNCA in CSF is present in an insoluble fraction or that it is modified in such a way that it no longer becomes recognizable by the antibodies employed in the sandwich assay, and this modifying factor needs to be resistant to the various denaturing treatments tested in this work as well as specific for CSF. This CSF matrix effect, if present, must be specific for pS129 SNCA and cannot generically affect detection of any phospho-protein in CSF as e.g., phosphorylated TAU proteins are readily detected in this matrix (Manniche et al., 2019; Seino et al., 2019). A final possibility is indeed that pS129 SNCA is not present in CSF, at least in the samples we analyzed; and indeed, the detection of pS129 SNCA levels in CSF remains somewhat controversial (Parnetti et al., 2019). Clearly, further work is required to explore matrix effects in CSF as well as the influence of SNCA modifications and conformation on the specificity and sensitivity of the different immunoassays so far developed for SNCA and its variants. Generally, as compared to other sandwich immunoassay platforms, Singulex assays can achieve higher sensitivity thanks to an analyte enrichment step and digital counting, which is particularly important when analyzing scarce analytes in limiting biological samples, as is often the case with clinical samples. The design of the assays presented in this manuscript, employing complementary pS129 SNCA and total SNCA measurements sharing the same capture antibody, enable normalization of the analyte of interest and therefore an accurate quantification of the proportion of the SNCA pool which is phosphorylated. As with all antibody-based assays, Singulex assays have limitations. Specifically, detection is based on epitope availability, which can be influenced by conformational confounders, protein-protein interactions and mutations/modifications affecting the epitopes interrogated by antibodies. For this reason, orthogonal validation using antibody-independent methodologies such as mass-spectrometry should be employed, though this may not always be possible due to sensitivity issues. Despite this, these novel SNCA and pS129 SNCA assays represent valuable and ultrasensitive tools to further explore the biology of SNCA and the development of therapeutics and diagnostics for synucleopathies.

## Data Availability

All datasets generated for this study are included in the manuscript and/or the Supplementary Files.

## Author Contributions

AC and LP designed the research. CC, PM, MV, LA, GB, and LP performed the research. AW, SD, HL, ES, GMS, TS, NM, and GS contributed reagents and samples. CC, PM, and LP analyzed the data. CC, AC, and LP wrote the manuscript. AW and HL revised the manuscript.

## Conflict of Interest Statement

GB and AW were employed by the company IRBM Promidis at the time of this study. The remaining authors declare that the research was conducted in the absence of any commercial or financial relationships that could be construed as a potential conflict of interest.
