# Supplementary figures and images for: Phospho-S129 Alpha-Synuclein Is Present in Human Plasma but Not in Cerebrospinal Fluid as Determined by an Ultrasensitive Immunoassay
Source: Front Neurosci. 2019 Aug 22;13:889. doi: 10.3389/fnins.2019.00889 (PMC6714598; doi:10.3389/fnins.2019.00889)

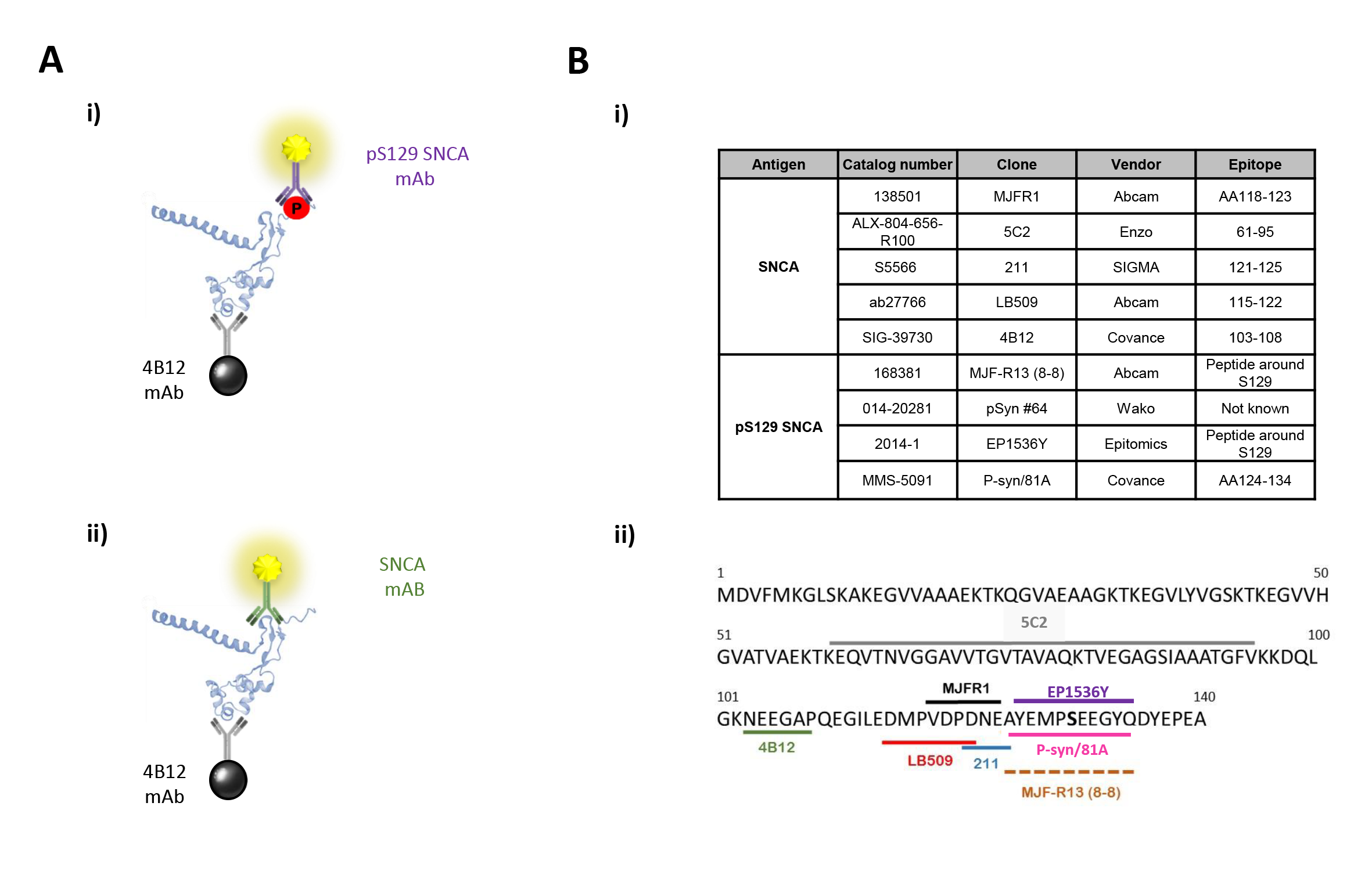

Supplement: Supplementary file 1 [file Image_1.TIF]

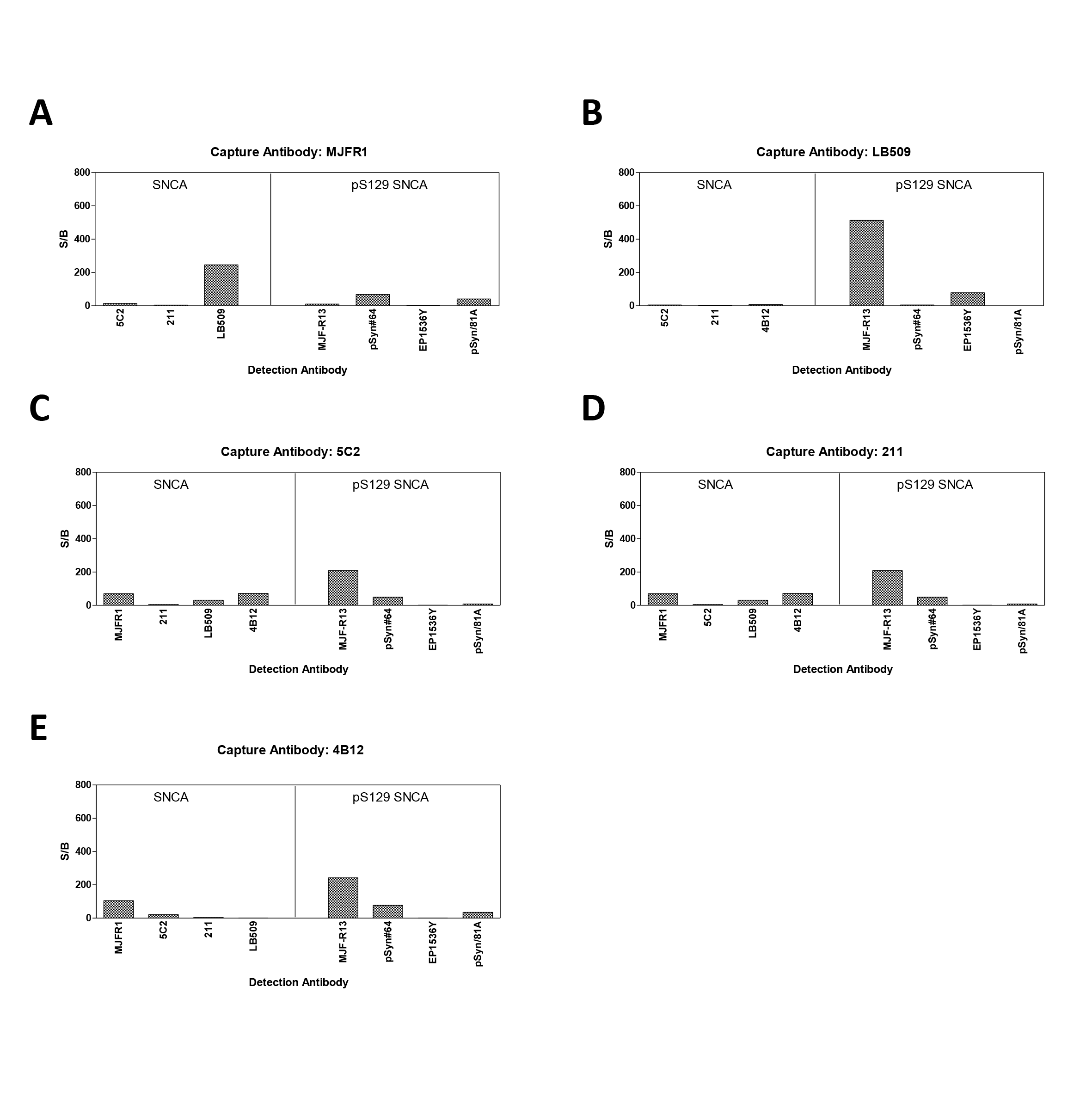

Supplement: Supplementary file 2 [file Image_2.TIF]

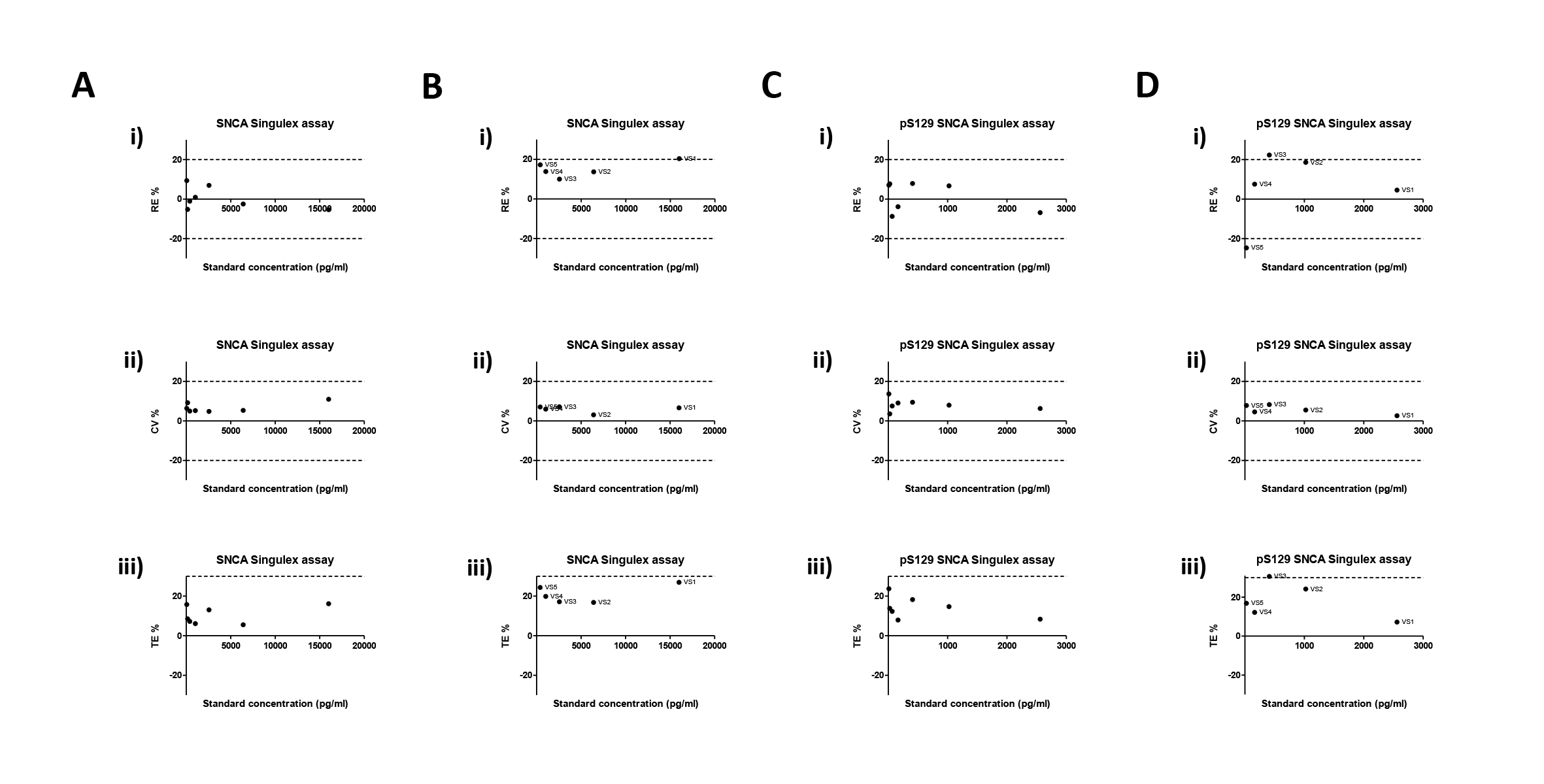

Supplement: Supplementary file 3 [file Image_3.TIF]

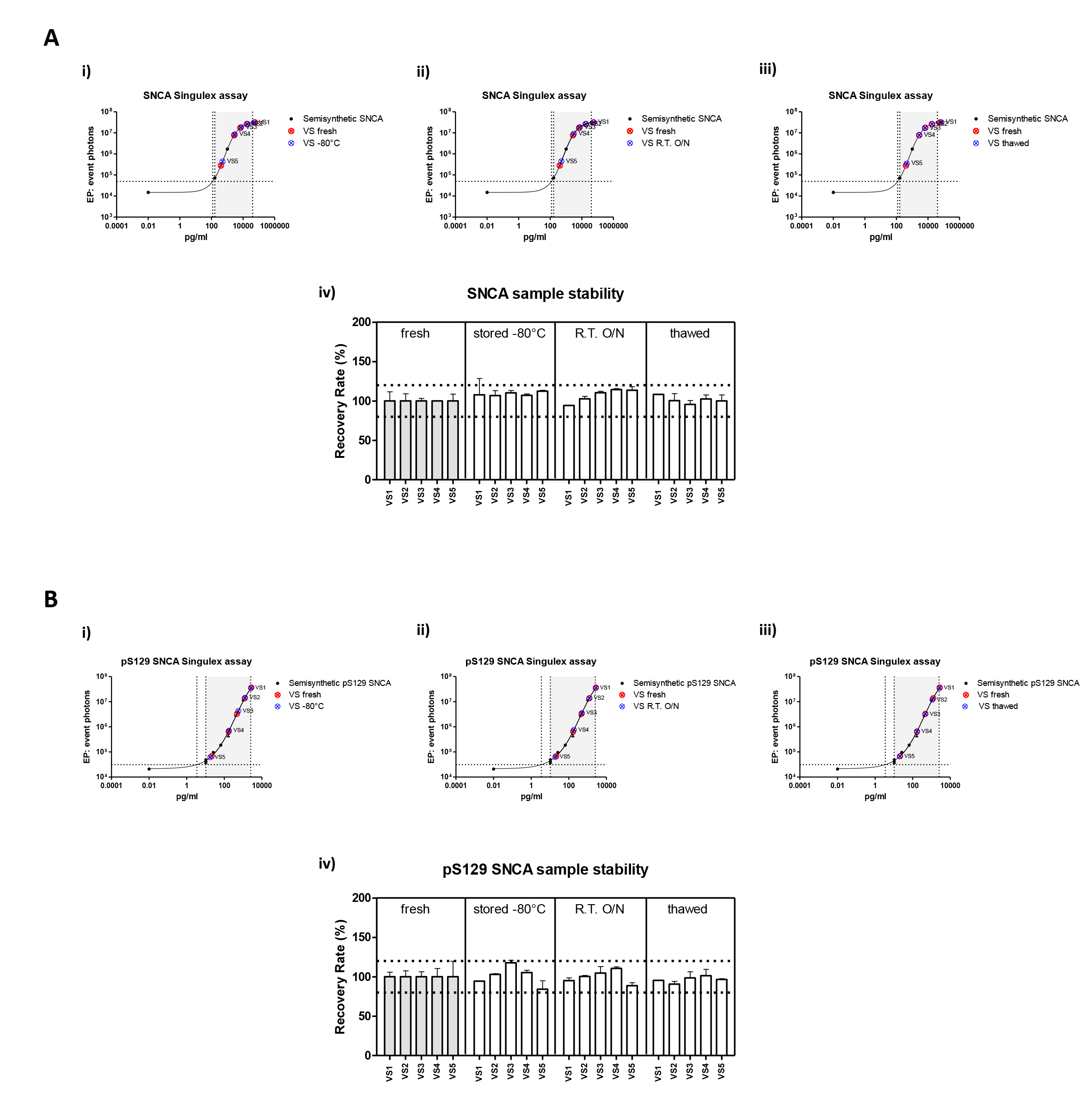

Supplement: Supplementary file 4 [file Image_4.TIF]

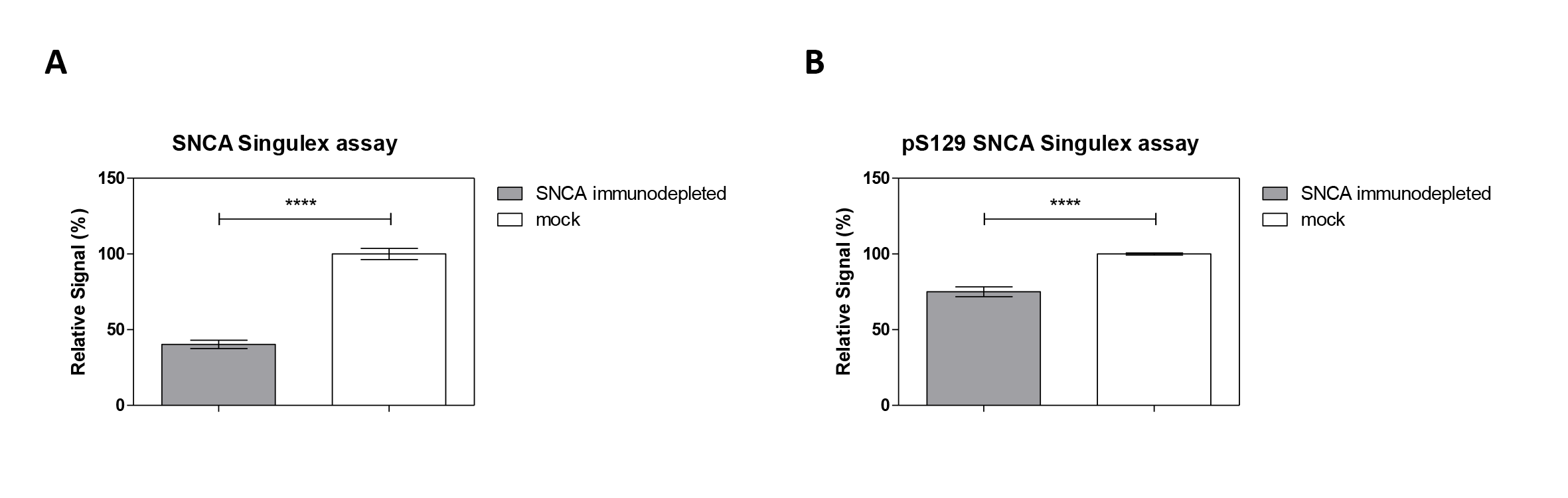

Supplement: Supplementary file 5 [file Image_5.TIF]
