# Supplementary material for: Phospho-S129 Alpha-Synuclein Is Present in Human Plasma but Not in Cerebrospinal Fluid as Determined by an Ultrasensitive Immunoassay
Source: Front Neurosci. 2019 Aug 22;13:889. doi: 10.3389/fnins.2019.00889 (PMC6714598; doi:10.3389/fnins.2019.00889)
Supplement: Supplementary file 6 [file Table_1.DOCX]

***Supplementary Material***

**Supplementary Figures**


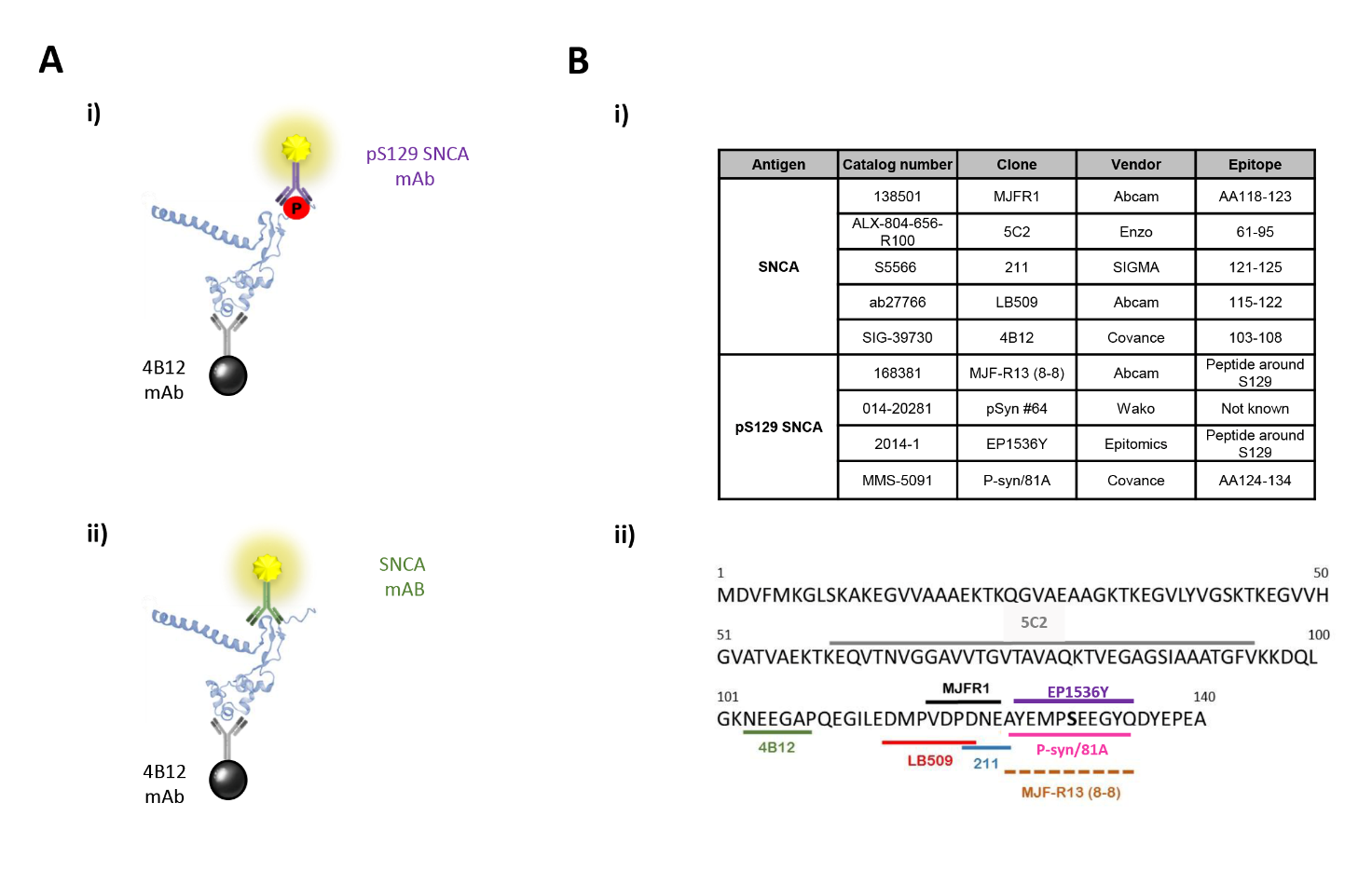


**Supplementary Figure 1. Development of Singulex assays for the detection of SNCA and pS129 SNCA**. **A**. Illustration of the approach for the detection of SNCA and its pS129 variant, with a single capture mAb and 2 detection mAbs specific for the pS129 epitope (i) or for an unrelated SNCA epitope (ii). **B** (i) Table of mAbs evaluated for SNCA and pS129 SNCA Singulex assays development and (ii) their epitope (where defined) within the SNCA protein sequence.

**Supplementary Figure 2. Combinatorial analysis of SNCA and pS129 SNCA antibodies in the Singulex assays, using purified semisynthetic SNCA and pS129 SNCA proteins. A.** Assays employing MJFR1 as SNCA capture antibody in combination of other anti-SNCA mAbs (for SNCA detection) or anti-pS129 SNCA mAbs (for pS129 SNCA detection). **B.** Assays employing LB509 as SNCA capture antibody in combination of other anti-SNCA mAbs (for SNCA detection) or anti-pS129 SNCA mAbs (for pS129 SNCA detection). **C.** Assays employing 5C2 as SNCA capture antibody in combination of other anti-SNCA mAbs (for SNCA detection) or anti-pS129 SNCA mAbs (for pS129 SNCA detection). **D.** Assays employing 211 as SNCA capture antibody in combination of other anti-SNCA mAbs (for SNCA detection) or anti-pS129 SNCA mAbs (for pS129 SNCA detection). **E.** Assays employing 4B12 as SNCA capture antibody in combination of other anti-SNCA mAbs (for SNCA detection) or anti-pS129 SNCA mAbs (for pS129 SNCA detection).

**Supplementary Figure 3.** **SNCA and pS129 SNCA Singulex assays performance.** **A.** Relative error (i), coefficient of variation (ii) and total error (iii) parameters of the SNCA Singulex assay using purified semisynthetic SNCA protein. **B.** Accuracy and precision of the SNCA Singulex assay carried out on validation samples (VS). VS1: artificial CSF spiked with 16000 pg/ml (ULOQ), VS2: dilution of 1:2.5 of VS1, VS3: dilution 1:2.5 of VS2, VS4: dilution 1:2.5 of VS3, VS5: dilution 1:2.5 of VS4. The VS samples were spiked independently from the calibrations standard, using separately prepared stock solutions and were analyzed against the calibration curve (respectively for each readout). The measurements were performed by two analysts across 6 independent assays runs over several days. **C.** Relative error (i), coefficient of variation (ii) and total error (iii) parameters of the pS129 SNCA Singulex assay using purified semisynthetic pS129 SNCA protein. **D.** Accuracy and precision of the pS129 SNCA Singulex assay carried out on validation samples (VS). VS1: artificial CSF spiked with 2560 pg/ml (ULOQ), VS2: dilution of 1:2.5 of VS1, VS3: dilution 1:2.5 of VS2, VS4: dilution 1:2.5 of VS3, VS5: dilution 1:2.5 of VS4. The VS samples were spiked independently from the calibrations standard, using separately prepared stock solutions and were analyzed against the calibration curve (respectively for each readout). The measurements were performed by two analysts across 6 independent assays runs over several days.

**Supplementary Figure 4.** **SNCA and pS129 SNCA Singulex assays validation**. **A.** Stability analysis of the SNCA Singulex assay signal, analyzed on validation samples (VS) described in S2B. Detection of freshly prepared VS is compared to the detection of VS stored at -80°C overnight (i), stored at room temperature overnight (ii) or subjected to a rapid freeze-thaw cycle (iii). (iv) The recovery of VS is complete in all conditions tested by SNCA Singulex assay. **B.** Stability analysis of the pS129 SNCA Singulex assay signal, analyzed on validation samples (VS) described in S2D. Detection of freshly prepared VS is compared to the detection of VS stored at -80°C overnight (i), stored at room temperature overnight (ii) or subjected to a rapid freeze-thaw cycle (iii). (iv) The recovery of VS is complete in all conditions tested by pS129 SNCA Singulex assay.

**Supplementary Figure 5. Specificity of the Singulex assays for the detection of SNCA and pS129 SNCA using HEK293T cells expressing SNCA and GRK1, demonstrated by immunodepletion with an anti-SNCA (S5566-Sigma) mAb or with the unrelated GFAP mAb (G3893-Sigma)**. **A.** Specificity of detection of the SNCA Singulex assay. **B.** Specificity of detection of the pS129 SNCA Singulex assay. Mean and SD of 3 independent experiments. T-test (p<0.0001).
